# Coexpression Analysis Reveals Dynamic Modules Regulating the Growth and Development of Cirri in the Rattans (*Calamus simplicifolius* and *Daemonorops jenkinsiana*)

**DOI:** 10.3389/fgene.2020.00378

**Published:** 2020-05-12

**Authors:** Jiongliang Wang, Xuelian Ma, Jiaotong Yang, Yanan Hui, Jiajie She, Tian Tian, Zhongqiu Li, Wenying Xu, Zhimin Gao, Zhen Su, Hansheng Zhao

**Affiliations:** ^1^State Forestry and Grassland Administration/Beijing Key Open Laboratory on the Science and Technology of Bamboo and Rattan, Institute of Gene Science and Industrialization for Bamboo and Rattan Resources, International Center for Bamboo and Rattan, Beijing, China; ^2^State Key Laboratory of Plant Physiology and Biochemistry, College of Biological Sciences, China Agricultural University, Beijing, China

**Keywords:** rattan, gene network analysis, gene coexpression, growth and development, *Calamus simplicifolius*, *Daemonorops jenkinsiana*

## Abstract

Rattan is regarded as one of the major non-timber forest products, second only to wood and bamboo, worldwide. Although the published genomes of *Calamus simplicifolius* and *Daemonorops jenkinsiana* have facilitated genome-wide gene functional analyses, coexpression networks (CENs) provide more comprehensive and complete annotations of gene function at the transcriptome level. Thus, we analyzed the CENs of the two rattans, *C. simplicifolius* and *D. jenkinsiana*, by integrating the genome sequences and analyzing in-house transcriptome data from different development stages of their cirri using a well-developed strategy. A total of 3,504 and 3,027 functional modules were identified in *C. simplicifolius* and *D. jenkinsiana*, respectively, based on a combination of CENs, gene family classification, and function enrichment tools. These modules covered the major developmental processes, including photosynthesis, lignin biosynthesis, flavonoid biosynthesis, and phenylpropanoid biosynthesis. Reference annotations were refined using CENs and functional modules. Moreover, we obtained novel insights into the regulation of cirrus growth and development in rattans. Furthermore, Rattan-NET (http://rattan.bamboogdb.org/), an online database with analysis tools for gene set enrichment analysis, module enrichment, network comparison analysis, and *cis*-element analysis, was constructed for the easy analysis of gene function and regulation modules involved in the growth and development of cirri in rattans.

## Introduction

Rattans are a major group of perennial flowering plants that are evergreen climbers, belonging to the subfamily Calamoideae (Jiang, 2007). More than 600 rattan species belonging to 13 genera have been identified. Of these, 27 species are used commercially and are cultivated extensively in tropical regions (Vorontsova et al., 2017). More than 5 million people rely on rattans, which generate approximately $7 billion in revenue each year (Kumar et al., 2012). Rattans also constitute an integral component of the tropical forest ecosystem. They are characterized by whip-like cirri containing hooks and grapnels that assist in climbing and development in a forest environment (Raj et al., 2014). However, in most cases, this makes it difficult to cultivate, manage, and harvest rattan, resulting in the high cost of rattan products (Zhao et al., 2017a). The genetic regulatory mechanism of cirrus formation is thus of great concern. Gene function annotations have significant implications in molecular biological studies and help researchers gain novel insights into the biological characteristics of organisms. In 2018, our team published two representative rattan genomes (*Calamus simplicifolius* and *Daemonorops jenkinsiana*) and their corresponding annotations as part of the Genome Atlas of Bamboo and Rattan (GABR) project (Zhao et al., 2017b, 2018). However, a transcriptome-level analysis of gene function has not been reported for rattans.

Gene cofunction networks play a crucial role in generating holistic pathway models (Rhee and Mutwil, 2014), refining gene annotations, and simulating significant regulatory mechanisms *in vivo* (Ma et al., 2018). Coexpression network (CEN) analysis is a powerful tool to characterize genes of related functions based on correlated transcriptional expression levels, which enables simultaneous annotation, clustering, and exploration of numerous genes under various conditions (Serin et al., 2016). Coexpression networks are widely used in gene function annotation, especially with the increase in the number of whole-genome sequences released, because they provide meaningful insights into functional linkages between gene pairs, and have the ability to recognize gene transcriptional regulatory mechanisms *in vivo* and integrate high-throughput transcription datasets on a genome-wide level (Aoki et al., 2016; You et al., 2017; Ma et al., 2018; Tian et al., 2018; She et al., 2019). Coexpression networks and their corresponding analytical platforms have been developed in plants. For example, ATTED-II, released in 2016 (Aoki et al., 2016), is a CEN database offering coexpression datasets for nine plant species (*Arabidopsis thaliana*, field mustard, soybean, barrel medic, poplar, tomato, grape, rice, and maize). CcNET is a CEN for comparative gene function analyses in multidimensional networks of diploid and polyploid *Gossypium* species (You et al., 2017). AraNet v2 (Lee et al., 2015b), an orthology-based CEN of non-model plant genes, provides network-assisted functional predictions for 28 plant species. RiceNet v2 (Lee et al., 2015a) is an improved network prioritization server for rice, which uses machine learning algorithms to integrate larger amounts of data and incorporate network analysis methods. A bamboo CEN, Bamboo-NET, mainly provides refined gene function annotations for *Phyllostachys edulis* (Ma et al., 2018). Although many of the constructed CENs have covered numerous model and non-model species (Ma et al., 2018; She et al., 2019), a rattan CEN has not yet been constructed.

Generally, the construction of a CEN follows three steps (Serin et al., 2016): calculating similarity scores between two genes, forming a network based on extracted gene pairs by selecting a similarity score threshold, and identifying modules from the network. There are multiple methods in each step, and therefore, different combinations are used in CEN construction. For example, the weighted correlation network analysis (WGCNA) package, a package for CEN analysis, provides alternative measures to assess coexpression similarity (Langfelder and Horvath, 2008). Weighted correlation network analysis determines the power operation soft thresholds to build an adjacency matrix and then uses the hierarchical cluster tree method to identify functional modules. However, many established methods including WGCNA are less suitable for assisting gene functional annotation of non-model species, because many genes of interest may be filtered out by non-customized or overly stringent parameters prior to network construction and module identification. Here, we report two CENs for two rattan species, *C*. *simplicifolius* and *D*. *jenkinsiana*, using their genome sequences and various in-house transcriptome data. We chose a well-developed strategy (You et al., 2016, 2017; Ma et al., 2018; Tian et al., 2018; Da et al., 2019; She et al., 2019) that maximizes the genome coverage and coexpressed module numbers to refine the functional annotations of the genes. Based on the CENs, we identified functional modules covering different developmental stages of cirri in rattans and refined their gene function annotations. The reliability of the gene function predictions was evaluated using several functional analysis tools, such as gene family classification, orthologous annotation, *cis*-element, and Gene Ontology (GO) analyses. Based on a data-mining system, we suggest the possible superiority of the flavonoid biosynthesis pathway compared to the lignin biosynthesis pathway during the growth and development of cirri in rattans. Moreover, Rattan-NET, a web server for rattan CENs, was built to help researchers browse and investigate the CEN and to provide potential functional modules and gene annotations online via meaningful information and powerful tools.

## Materials and Methods

### Sample Description

For comprehensive network construction, samples of cirri from three developmental stages were collected from *C. simplicifolius* and *D. jenkinsiana* (Supplementary Table S1) in the spring of 2015, at the Research Institute of Tropical Forestry of the Chinese Academy of Forestry in the city of Guangzhou, Guangdong Province, China (N: 23°11′29′′, E: 113°22′40′′, 87 m). TRIzol Reagent (Invitrogen, Carlsbad, CA, United States) was used to isolate RNA, according to the manufacturer’s instructions, and a NanoDrop 2000 spectrophotometer (Thermo Fisher Scientific, Waltham, MA, United States) was used to determine the RNA purity and concentration. RNA was reverse transcribed using a reverse transcription system (Promega, Madison, WI, United States). As described previously (Zhao et al., 2017a), the extracted RNA was treated with RNase-free DNase I for 30 min at 37°C to remove residual DNA before reverse transcription. The pooled libraries were then sequenced using a BGISEQ-500 platform (Beijing Genomics Institute, Shenzhen, China), with 100-bp paired-end reads. Furthermore, four additional in-house samples of cirri were collected from *D. jenkinsiana*, which had previously been sequenced by RNA-seq (Zhao et al., 2017a). Thus, a total of 24 and 36 in-house transcriptome datasets, covering different developmental stages of cirri, were processed for *C. simplicifolius* and *D. jenkinsiana*, respectively. Datasets from the same tissue were merged for subsequent analyses.

### Data Processing and Gene Expression Profile Analysis

For data preprocessing, all transcriptomic datasets were analyzed by FastQC v0.11.6 (Brown et al., 2017), with the default parameters for a statistical analysis of quality. Trimmomatic v0.36 (Bolger et al., 2014) was used to filter the adapters and low-quality sequences using the following parameters: LEADING:3, TRAILING:3, SLIDINGWINDOWS 4:15, MINLEN:50, and TOPPHRED64. For data mapping, clean data from Trimmomatic were mapped to the respective reference genomes (Zhao et al., 2018) using HISAT2 v2.1.0 (Kim et al., 2015) software, with the following modifications from the default parameters: –min-intronlen 20, –max-intronlen 4000, and –rna-strandness RF. All datasets from HISAT2 were reserved (Supplementary Table S2) for transcript assembly. During transcript assembly, the fragments per kilobase of transcript per million mapped reads (FPKM) values were obtained using Cufflinks v2.2.1 (Trapnell et al., 2012), with the default parameters, except for an additional parameter,-u. Differentially expressed genes were assessed using Cuffdiff v2.2.1 (Trapnell et al., 2012) using the default parameters. Additionally, the 3σ criterion formula of “threshold = average (5% value) + 3^∗^ SD” (You et al., 2017) was used to calculate the FPKM thresholds in each experimental group (Supplementary Figure S1). The genes with FPKM values below the threshold in each sample were removed because of insignificant correlation coefficients. The FPKM threshold values were 0.27 and 0.18 for *C. simplicifolius* and *D. jenkinsiana*, respectively, as shown in Supplementary Figure S1.

### CEN Construction

As previously mentioned, a well-developed strategy (You et al., 2016, 2017; Ma et al., 2018; Tian et al., 2018; She et al., 2019) combined with Pearson correlation coefficients (PCCs) and mutual ranks (MRs) was applied to construct rattan CENs (Supplementary Figure S2). First, we calculated PCC values of gene pairs based on the FPKM values of the genes. We removed weakly correlated gene pairs, and only those with strong correlation were reserved (Supplementary Figure S3). Second, we calculated MR values of genes based on PCC values. The MR served as the geometric average of the PCC ranks from gene A to gene B and from gene B to gene A, and it was used to extract high-confidence gene pairs and eliminate less reliable gene pairs. Receiver operating characteristic analysis, based on the distribution of MR values, was performed to determine the optimal parameters for MR analysis. Finally, coexpression gene pairs with a unidirectional rank less than 3 (*Rank*[*A*→*B*] or [*B*→*A*]) and an MR value less than 30 were collected.

PCC algorithm:

$$P\,C\,C=\frac{\sum_{i=1}^{n}\left(x_{i}-\bar{x}\right)\,\left(y_{i}-\bar{y}\right)}{\sqrt{\Sigma_{i=1}^{n}\,{\left(x_{i}-\bar{x}\right)}^{2}\cdot \Sigma_{i=1}^{n}\,{\left(y_{i}-\bar{y}\right)}^{2}}}$$

In the PCC algorithm, *X* and *Y* are FPKM values, and *n* represents the number of samples.

MR algorithm:

$$M\,R\,\left(A\,B\right)=\sqrt{\left(Rank\left(A\to B\right)\cdot Rank\left(B\to A\right)\right)}$$

In the MR algorithm, *Rank*(*A*→*B*) is the PCC rank from gene A to gene B and *Rank*(*B*→*A*) is the PCC rank from gene B to gene A.

### Functional Module Identification and Gene Annotation Refining

CFinder v2.0.6 (Adamcsek et al., 2006), based on the clique percolation method (CPM), was applied to identify modules by measuring the node density of the CENs in the rattans. To maximize gene coverage and module number, *k* = 6 and *k* = 5 were selected for *C. simplicifolius* and *D. jenkinsiana*, respectively, according to Supplementary Figure S4. These parameters optimized the coverage for both module counts and gene functional annotation. For example, *k* = 5 indicates that a module had five or more nodes. In addition, the function of the modules was annotated by gene set enrichment analysis (GSEA) (Yi et al., 2013) based on multiple types of function terms, including plant ontology (PO), GO, GFam (for gene families), and Kyoto Encyclopedia of Genes and Genomes (KEGG) terms. Suspected modules were filtered by Fisher exact test and multiple hypothesis testing. Gene functional annotation refining is an imperative application of a CEN. We refined the gene functional annotation with modules and CENs. First, the genes belonging to the modules were annotated with the module functional annotations. Second, we performed GSEA using coexpression gene sets of each gene, and the results with a false discovery rate (FDR) < 0.05 were recognized as refined annotations.

### Identification of Orthologous Genes Between Rattans and *A. thaliana*

Orthologous identification was performed based on the method of reciprocal best hit (RBH) BLAST (Moreno-Hagelsieb and Latimer, 2008), to identify potential orthologous proteins between the rattans and *A. thaliana*. The top three hits of each RBH were identified as the best orthologous pairs. Pairs of *E* values less than the peak of the *E*-value distribution of all the best hits were identified as the thresholds of secondary orthologous pairs in *C. simplicifolius* and *A. thaliana*, *D. jenkinsiana* and *A. thaliana*, and *C. simplicifolius* and *D. jenkinsiana*.

### Significance Analysis for *Cis*-Elements

Previously described filtering strategies of the *Z* score and *P* value were used in the *cis*-element significance test (Endo et al., 2014). The motifs with a *P* < 0.05 were selected as significantly enriched regulatory modules when scanning the 3-kb promoter regions of rattan genes.

The *Z* score was calculated as follows:

$$Z=\frac{\bar{X}-u}{\sigma /\sqrt{n}}$$

where $\bar{X}$ is the sum value of a motif in the promoter of one gene list; *u* is the mean value of the same motif in 1,000 random genes with the same scale; and σis the standard deviation of the 1,000 random genes.

### Identification and Phylogenetic Analysis of the Light-Harvesting Complex Gene Families

The sequences of *A. thaliana* light-harvesting complex (*LHC*) were downloaded from Tair^1^ (accessed May 2019). All sequences derived from Tair were used as queries to search against datasets for three species using BLAST+, with an *e*-value cutoff of 1e-05. The five datasets included those for *C. simplicifolius*, *D. jenkinsiana*, and *Oryza sativa*. The genome resource of *O. sativa* was downloaded from phytozome11^2^ (accessed May 2019). The results were then examined by domain analysis using the Perl script pfamscan.pl (Li et al., 2015), and the coding sequences of the five gene families were extracted.

Subsequently, we performed multiple sequence alignment using MUSCLE v3.8.31 (Edgar, 2004) with the default parameters. Conserved sequences were extracted using the Gblocks server (Talavera and Castresana, 2007)^3^. jModeltest v2.1.6 (Darriba et al., 2012) was then used to search the best PhyML model in the conserved sequences, with the following parameters: -f, -g 4, -i, -s 203, -S BEST. Finally, phylogenetic trees were constructed using the software PhyML v20120412 (Guindon et al., 2010) and the model suggested by jModeltest. Tree topology was assessed by bootstrap analysis with 1,000 resampling replicates.

### Search and Visualization Platform Construction

The platform was constructed under the LAMP (Linux, Apache, Mysql, and PHP) environment. Calculations performed by analysis tools were based on Python, Perl, and R scripts. Cytoscape.js, the java version of Cytoscape in Linux, was used to construct functional modules and for CEN dynamic display. GBrowse (Stein, 2013) and SequenceServer (Priyam et al., 2019) were used to provide sequence scanning and BLAST+ tools in rattans.

## Results

### Construction of CENs and Identification of Potential Functional Modules

We processed 24 and 36 in-house transcriptome datasets covering different developmental stages of cirri from *C. simplicifolius* and *D. jenkinsiana*, respectively, to construct the rattan CENs (Supplementary Table S1). The construction strategy was well-developed and has been widely used (You et al., 2017; Ma et al., 2018; Tian et al., 2018; She et al., 2019). A total of 630,081 and 670,502 gene pairs with the following criteria: top 3 MRs + MR ≤ 30, were identified as CEN edges in *C. simplicifolius* and *D. jenkinsiana*, respectively (Table 1). The CENs contained 31,847 nodes covering 62.16% of *C. simplicifolius* genes and 36,769 nodes covering 68.93% of *D. jenkinsiana* genes (Table 1). The mean edges in the two rattan networks were similar (Table 1). The density plot of network edges indicates that only a small number of genes are highly connected, consistent with general feature of biological networks (Supplementary Figure S5A).

**TABLE 1:** Coexpression network statistics.

Rattan species	Coexpression network			Module	
		
	Coexpression pairs	Network nodes	Mean edges of gene	Number	Mean nodes
*Calamus simplicifolius*	630,081	31,847	39.6	3,504	8.1
*Daemonorops jenkinsiana*	670,502	36,769	36.4	3,027	6.1

We then identified potential functional modules in the rattans using CFinder, based on the CPM and GSEA, with an FDR cutoff value < 0.05. There were 3,504 and 3,027 potential functional modules identified in *C. simplicifolius* and *D. jenkinsiana*, respectively (Table 1), and the distribution map showed that the number of modules decreased as the number of genes per module increased (Supplementary Figure S5B). The mean nodes of the modules were 8.1 and 6.1 in *C. simplicifolius* and *D. jenkinsiana*, respectively (Table 1). The modules annotated by GSEA with PO, GFam, GO, and KEGG gene sets indicated that the majority of biological processes during development were covered, including photosynthesis, plant type, secondary cell wall (SCW) biogenesis, the lignin biosynthetic process, flavonoid biosynthesis, and phenylpropanoid biosynthesis. We further analyzed the significant GO terms (*Z* score > 4) in the modules of the two rattans (Supplementary Table S3). Some conserved terms were found between the two rattans, including ubiquitin-dependent protein catabolic process, proton transmembrane transport, vesicle-mediated transport, and response to cytokinin.

The annotations generated by GSEA were provided on the Download page of Rattan-NET. In summary, 54.6 and 62.4% of genes were annotated following the CEN and module analyses for *C. simplicifolius* and *D. jenkinsiana*, respectively.

### CEN Analysis of the Flavonoid and Lignin Biosynthesis Pathways in the Rattans

**FIGURE 1:**
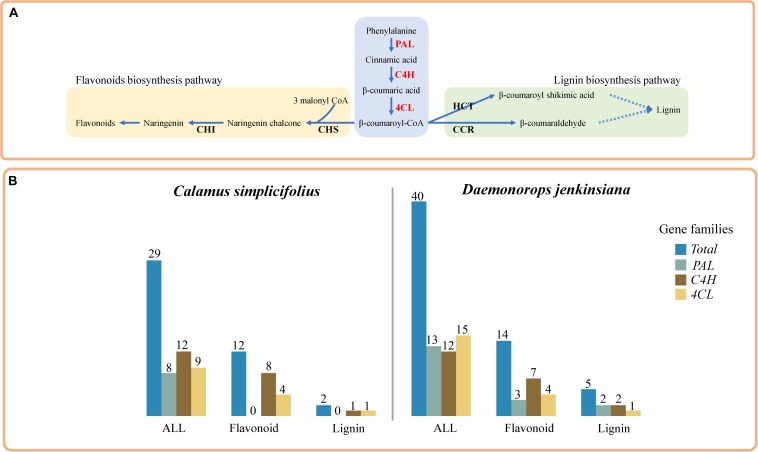
Comparative analysis of lignin and flavonoid biosynthesis pathways in rattans. **(A)** Sketch of the lignin biosynthesis and flavonoid biosynthesis pathways. **(B)** Bar plot of the flavonoid biosynthesis–related gene counts and lignin biosynthesis–related coexpression gene counts in the *4CL*, *C4H*, and *PAL* families of *C. simplicifolius* and *D. jenkinsiana*. In total, there are 29 and 40 members of the *PAL*, *C4H*, and *4CL* families in *C. simplicifolius* and *D. jenkinsiana*, respectively.

We used RBH to identify *PAL*, *C4H*, and *4CL* genes, which are involved in phenylpropanoid pathway (Figure 1A) (Li et al., 2010), in the rattans. There are 8, 12, and 9 gene members of *PAL*, *C4H*, and *4CL* gene families in *C. simplicifolius*, respectively (Supplementary Table S4). The corresponding numbers are 13, 12, and 15 in *D. jenkinsiana*. Then, we investigated the coexpressed flavonoid biosynthesis–related and lignin biosynthesis–related genes in the *PAL*, *C4H*, and *4CL* gene families in rattans. Functional annotation terms of lignin biosynthesis and flavonoid biosynthesis were detected in these coexpressed genes, including a total of 29 and 40 genes from the *PAL*, *C4H*, and *4CL* families in *C. simplicifolius* and *D. jenkinsiana*, respectively (Figure 1B). The number of flavonoid biosynthesis–related genes in the *PAL*, *C4H*, and *4CL* families was 12 and 14 in *C. simplicifolius* and *D. jenkinsiana*, respectively (Figure 1B and Supplementary Table S5). Compared to the number of flavonoid biosynthesis–related genes, low numbers of lignin biosynthesis–related genes (2 in *C. simplicifolius* and 5 in *D. jenkinsiana*) were identified in the *PAL*, *C4H*, and *4CL* families (Figure 1B). The results showed that more flavonoid biosynthesis–related genes than lignin biosynthesis–related genes are present in the *PAL*, *C4H*, and *4CL* gene families in rattans.

### CEN Analysis of Secondary Cell Wall Biosynthesis in the Rattans

**FIGURE 2:**
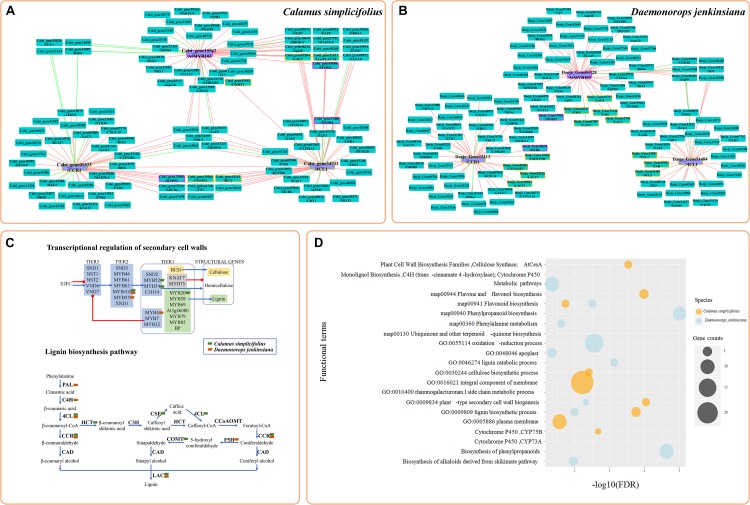
Coexpression network analysis of the genes involved in secondary cell wall biosynthesis in rattans. **(A)** The coexpression network (CEN) of *Calsi_gene34733* (*4CL1*), *Calsi_gene01533* (*CCR1*), and *Calsi_gene15542* (*MYB103*) in *C. simplicifolius*. **(B)** The CEN of *Daeje_Gene51484* (*4CL1*), *Daeje_Gene15213* (*CCR1*), and *Daeje_Gene04728* (*MYB103*) in *D. jenkinsiana.* **(A,B)** The red and green lines link the positive and negative coexpression gene pairs, respectively. The rectangles with purple and yellow borders represent gene families involved in the transcriptional regulation of secondary cell walls (SCWs) and the lignin biosynthesis pathway, respectively. **(C)** Transcriptional regulation of SCW and lignin biosynthesis pathways (Kumar et al., 2016). The green and orange blocks indicate genes in the coexpression network of **(A,B)**, respectively. Transcription factors (TFs) involved in SCW biosynthesis can be broadly classified into three groups. Tier 1 TFs bind directly to the structural genes responsible for the biosynthesis of cell wall components. Tier 2 TFs can regulate Tier 1 TFs in addition to structural genes, whereas Tier 3 TFs can regulate Tier 2 TFs and the structural genes. The blue arrows indicate positive regulation, whereas the red lines indicate negative regulation. Groups of TFs are shaded as follows: light green, lignin; light yellow, cellulose; others, light blue. The simplified pathway represents the most common pathways for the biosynthesis of lignin units. **(D)** GSEA results of coexpressed genes in **(A,B)**. The gene sets of GO, KEGG, and Gene Families (GFam) were selected.

We selected representative genes involved in the lignin biosynthesis pathway and in the transcriptional regulation of SCW biosynthesis (Hao and Mohnen, 2014; Kumar et al., 2016) and constructed their CENs (Figures 2A,B). These genes included *4CL1* (*Calsi_gene34733* and *Daeje_Gene51484*), *CCR1* (*Calsi_gene01533* and *Daeje_Gene15213*), and *MYB103* (*Calsi_gene15542* and *Daeje_Gene04728*) genes. The results showed that the transcripts of genes involved in SCW biosynthesis (Kumar et al., 2016), including *MYB20*, *MYB54*, and *MYB52* in *C. simplicifolius* and *MYB55* and *MYB4* in *D. jenkinsiana*, were identified in the SCW CENs and were mapped into a simplified sketch of the transcriptional regulation of SCWs (Figures 2A–C). Because lignin is one of the most significant components of the SCW, second only to cellulose, we detected many genes of the lignin biosynthesis pathway in the SCW CENs, and these genes were mapped to the lignin biosynthesis pathway (Figures 2A–C). Additionally, genes for irregular xylem (IRX), a core component of the SCW cellulose synthase complex (Kumar et al., 2016), were identified in the SCW CEN in *C. simplicifolius*. These included *IRX3* (*Calsi_gene01341*, *Calsi_gene30420*), *IRX1* (*Calsi_gene32935*), and *CESA4* (*Calsi_gene08952*). Meanwhile, *IRX6* (*Daeje_Gene45742*), *IRX3* (*Daeje_Gene59357*), and *CESA4* (*IRX5*, *Daeje59357*) were also found in the SCW CEN in *D. jenkinsiana* (Figures 2A,B). Through motif analysis of the 3-kb *4CL1* and *CCR1* gene promoter regions, the “MYB61” element was found to be significantly enriched (*P* < 0.05, Supplementary Table S6).

To discover network-based potential bioprocesses, we performed GSEA of the CENs of *C. simplicifolius* and *D. jenkinsiana* using the GO, KEGG, and GFam gene datasets (Figure 2D). The terms plant type SCW biogenesis (GO:0009834), lignin biosynthetic process (GO:0009809), and flavonoid biosynthesis (map00941) were enriched in both *C. simplicifolius* and *D. jenkinsiana*, suggesting the conservation of these bioprocesses in the two rattans. Additionally, the GO term cellulose biosynthetic process (GO:0030244) and the KEGG term flavone and flavanol biosynthesis (map00944) were uniquely enriched in *C. simplicifolius*. The GO term lignin catabolic process (GO:0046274), the KEGG term phenylpropanoid biosynthesis (map00940), and the GFam term biosynthesis of phenylpropanoids were enriched only in *D. jenkinsiana*.

The coexpression analysis of rattans also identified the potential functional modules related to SCW biosynthesis (Supplementary Figure S6). Some functional terms were enriched in the modules after GSEA, with an FDR cutoff value < 0.05. These included cellulose biosynthetic process (GO:0030244), plant cell wall biosynthesis families, cellulose synthase-like, lignin biosynthetic process (GO:0009809), and cell plate formation, which are involved in plant-type cell wall biogenesis (GO:0009920).

### Photosynthesis-Related Gene Analysis in the Rattans

**FIGURE 3:**
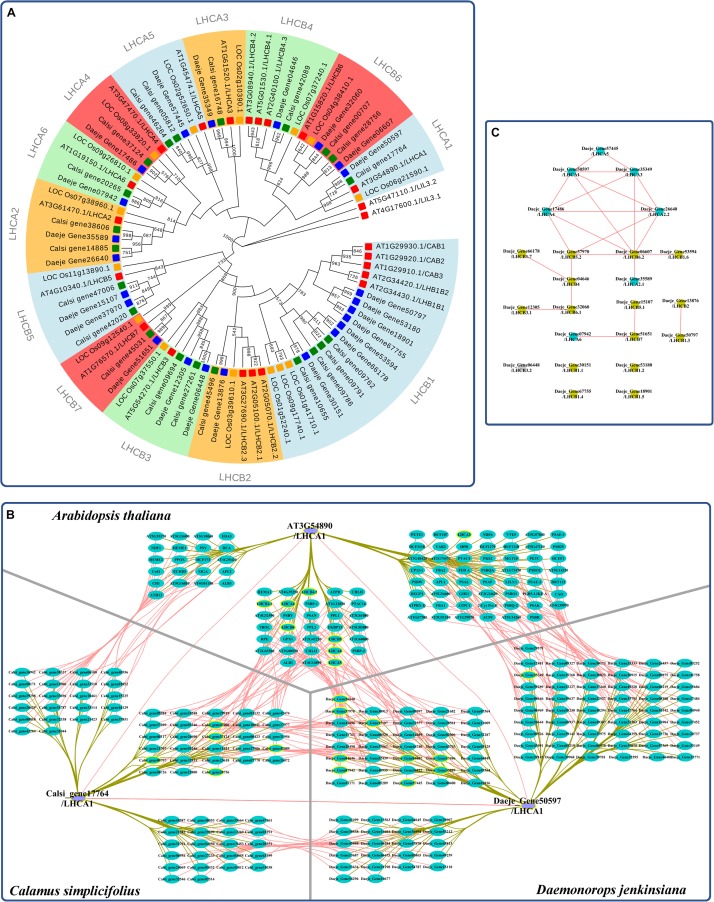
Coexpression analysis of *LHC* genes in the rattans. **(A)** Phylogenetic tree of the *LHC* gene family. The number at the node indicates the bootstrap value. *LILs* were selected as the outgroup. Green, blue, yellow, and red indicate the genes in *C. simplicifolius*, *D. jenkinsiana*, *O. sativa*, and *A. thaliana*, respectively. **(B)** Comparison of genes with the top 300 Pearson correlation coefficient (PCC) values of *LHCA1* in *C. simplicifolius*, *D. jenkinsiana*, and *A. thaliana.* The gray and dark yellow lines link the orthologous gene pairs and coexpressed genes, respectively. *LHC* genes are bordered with a yellow line. **(C)** The coexpression network between *LHC* genes in *D. jenkinsiana.* The blue and yellow dots are *LHCA* and *LHCB* genes, respectively. The gray dot indicates that there is no coexpression network for the gene. Red lines link the coexpressed gene pairs and indicate a positive coexpression relationship.

We reidentified the LHC genes and reconstructed a genome-scale phylogenetic tree to investigate the evolution of the *LHC* family in rattans (Figure 3A and Supplementary Table S7). The phylogenetic tree showed that the *LHC* gene family was divided into 13 subfamilies (Figure 3B). There were 21 and 23 *LHC* genes in *C. simplicifolius* and *D. jenkinsiana*, respectively, which was similar to the 21 *LHC* members in *A. thaliana* and more than 15 *LHC* members in *O. sativa*. Although the total number and the number of the largest member of the subfamily (*LHCB1*) were similar in the rattans and *A. thaliana*, other subfamilies showed different distributions of the gene members. For example, the rattans had double the number of members in the *LHCB6*, *LHCA2*, *LHCB5*, and *LHCB3* subfamilies compared to that in *A. thaliana*.

We then analyzed the coexpressed genes of the *LHC* family in rattans by GSEA (Supplementary Figure S7). The result indicated that photosynthesis-related terms, such as photosynthesis (GO:0015979), chloroplast thylakoid membrane (GO:0009535), and response to light stimulus (GO:0009416), were significantly enriched (FDR < 0.05). This result was consistent with previous studies showing that the major function of LHC proteins is light harvesting, which provides energy for photochemical reactions (Pan et al., 2019). It also indicated the robustness and credibility of the rattan CENs. Moreover, a high degree of consistency was found by comparing the top 300 coexpressed genes of *LHCA1* in the rattans and *A. thaliana* based on their PCCs (collected from ATTED-II and AraNet) to determine the possible functional modules (Figure 3B). Many genes orthologous to *LHC* were detected in the comparison network, which also indicated the reliability of the rattan CENs. The CEN of *D. jenkinsiana LHC* genes was constructed to determine the potential cooperative relationship between *LHC* genes in photosynthesis during the developmental stages of cirri (Figure 3C). Based on the constructed network, most *LHC* genes were closely related, with the exception of four isolated genes (*Daeje_Gene30151*, *Daeje_Gene53180*, *Daeje_Gene67755*, and *Dae_Gene18901*) belonging to the *LHCB1* subfamily.

### Rattan-NET: An Online CEN Database for Rattans

We constructed the Rattan-NET database^4^ to facilitate gene function analyses of rattans based on CENs. Rattan-NET integrates the CEN analysis, *cis*-element analysis, GSEA, GBrowse (Stein, 2013), and Sequenceserver (Priyam et al., 2019) and will help researchers refine the annotation of rattan genes at the transcriptome level. The Cytoscape web tool was applied to provide an interactive screen of the CEN and the modules, and users are able to search for single or multiple genes in the CEN. Rattan-NET provides gene ID and keyword searches, based on authoritative database terms (Supplementary Table S8) such as GO, KEGG, and Gfam terms. The FPKM values for different tissues are provided in detail in each page for every individual gene. Moreover, GBrowse offers genome-structure scanning and sequence-extracting tools. Rattan-NET provides comprehensive datasets and convenient tools for users and is a continually optimizable platform, with expanding analysis tools, rattan species, and datasets.

## Discussion

Our team published two rattan genomes (*C. simplicifolius* and *D. jenkinsiana*) and their genome-wide gene annotations in 2018, which provided sufficient data to support molecular studies (Zhao et al., 2018). However, gene function annotation was lacking at the transcriptome level. Here, we introduced transcriptome information into the gene function annotation and constructed rattan CENs using previously described strategies (You et al., 2017; Ma et al., 2018; Tian et al., 2018; She et al., 2019). The reliability of the network was validated at multiple levels, based on different functional analyses, including orthologous gene identification, gene family classification, and GO analysis. The two networks were found to be scale-free networks (Supplementary Figure S5A), which indicated their reliability. In rattan CENs, 3,504 and 3,027 modules covering multiple biological processes critical for growth of cirri and the development of rattans were annotated by GSEA in *C. simplicifolius* and *D. jenkinsiana*, respectively. The GO terms conserved between the two rattans, including ubiquitin-dependent protein catabolic process, proton transmembrane transport, vesicle-mediated transport, and response to cytokinin, may indicate that these processes are conserved in the two rattans. However, the networks covered 62.16 and 68.93% of *C. simplicifolius* and *D. jenkinsiana* genes, respectively, and showed relatively lower coverage than the CEN network of *P. edulis* (>90%) (Ma et al., 2018). The comparison analyses of CENs and genome quality suggested that this lower coverage was due to the limited number of samples and the scattered scaffolds of *C. simplicifolius* and *D. jenkinsiana* (Zhao et al., 2018), compared to *P. edulis*. Based on the ongoing maintenance of the genomes of bamboo and rattan and the continuous development of the GABR project by our team (Zhao et al., 2017b), higher-quality assemblies, genome annotations, and more transcriptome datasets will be obtained from various samples of rattans, to provide more comprehensive data support for future network constructions and analyses. Meanwhile, CENs from other rattan species will also be constructed to perform comparative CEN analysis.

The lignin and flavonoid biosynthesis pathways are important to the rattans. Flavonoids are widely distributed in foods and beverages of plant origin, such as fruits, vegetables, and tea, and they have been shown to have medicinal value (Tohge et al., 2013). Rattans not only have advantages with regard to being sources of wood for furniture or handicraft production, but also have edible and medicinal value (Jiang, 2007). For example, *D. jenkinsiana* is used as a traditional medicine in China. “Dragon’s blood,” an exudate from *Daemonorops*, contains a pair of complex flavonoid trimers and has antiviral and anticancer effects and shows activity against osteoporosis, diabetes, inflammation, and platelet aggregation (Jura-Morawiec and Tulik, 2016; Schmid and Trauner, 2017). The lignin and flavonoid biosynthesis pathways share common steps in the general phenylpropanoid pathway and are shaped by relative competition (Li et al., 2010). Previous studies have shown that there are distinct PAL and 4CL isoforms suited for both the lignin and flavonoid biosynthesis pathways or for each one of these pathways in plants (Baldi et al., 2017; Lavhale et al., 2018). Thus, we compared the CENs of the *PAL*, *C4H*, and *4CL* gene families (Figure 1B). More flavonoid biosynthesis–related genes than lignin biosynthesis–related genes were found for the *PAL*, *C4H*, and *4CL* gene families in rattans. These results indicated the presence of more PAL, C4H, and 4CL isoforms suited for the flavonoid biosynthesis pathway than for the lignin biosynthesis pathway in rattans. It demonstrated the application of CEN to better understand the regulation of the pathways particularly important to rattans.

Secondary cell walls play an essential role in providing mechanical support to plants (Kumar et al., 2016) and are important factors in determining the properties of wood. Lignin is the second-most abundant polymer after cellulose and is present in the SCW of all vascular plants (Liu et al., 2018). In our study, we performed a CEN-based analysis of the *4CL1*, *CCR1*, and *MYB103* genes in rattans and identified many modules related to the SCW biosynthesis pathway (Figure 2 and Supplementary Figure S6). The promoters of *4CL1* and *CCR1* genes shared the “MYB61” element recognized by MYB61, which is involved in the regulation of xylem formation and SCW biosynthesis in *A. thaliana* (Romano et al., 2012; Kumar et al., 2016). The results showed the presence of many genes involved in regulating SCW and lignin biosynthesis pathways, which also demonstrated the reliability of the networks. We also identified some modules related to SCW biosynthesis. The CENs and modules identified potential regulatory elements for SCW biosynthesis, which may be beneficial for the genetic improvement of rattan wood properties.

Light may be the most important factor that influences growth in rattan plantations (Jiang, 2007). Therefore, it is necessary to study the light-harvesting process for potential molecular plant breeding applications in rattans. Photosynthesis can convert solar energy into chemical energy that is stored as organic matter (Pan et al., 2019), which, in turn, serve as raw materials for biological metabolic processes (Vogt, 2010). Light harvesting, which uses LHC, is the first and most significant step of photosynthesis (Pan et al., 2019). We found that the number of *LHC* members in rattans and *A. thaliana* was similar, but the two species showed differences in the distribution of *LHC* members in different families, which may indicate different evolutionary processes of *LHC*. LHC proteins tend to form polymers *in vivo* and *in vitro* (Pan et al., 2019). Polymers of LHCA1-LHCA4 and LHCA2-LHCA3 have been reported (Pan et al., 2019), which matches the tight coexpression relationship between *LHCA* genes observed in *D. jenkinsiana* (Figure 3C). However, the four isolated *LHCB1* genes separated with other *LHC* genes in the CEN, which may indicate their relative independence from other *LHC* genes in the photosystem during the development of cirri. Thus, the CEN of *LHCs* may contribute to the discovery of a coordinated mechanism between LHC proteins.

Providing a rattan database comprising genomic and transcriptomic resources and analytical platforms facilitates the investigation of the molecular biology of rattans. However, no such database was reported thus far. Therefore, we constructed Rattan-NET, including CENs, functional modules, genomic resources, and gene annotations (GO, KEGG, Pfam). Researchers can use this search tool to analyze the genes and CENs of their interest. Analytical tools such as GSEA and module enrichment tools were also integrated into Rattan-NET. For network display, Cytoscape was embedded into Rattan-NET. Therefore, Rattan-NET facilitates an increased understanding of gene function in rattans.

## Conclusion

Here, we constructed CENs of two rattan species and refined their gene function annotations by the integration of their genome sequences and using in-house transcriptome data from different developmental stages of their cirri and a previously described strategy. To evaluate the reliability of the gene annotations and predictions, functional enrichment tools, gene family classification, *cis*-element analysis, and GO analysis were used. This study yielded novel insights into the genetic basis of important agronomic traits through data-mining systems, and further insights into other significant traits may be revealed using a similar strategy. Moreover, an online database of rattan CENs, Rattan-NET, was constructed to facilitate the application of the CENs. The CENs constructed here will facilitate molecular analyses and improve our understanding of the molecular regulatory mechanisms of the important characteristics of rattans.

## Data Availability Statement

The transcriptome reads derived from multiple tissues have been uploaded and deposited in the European Nucleotide Sequence Archive (EMBL-EBI) with the project accession numbers PRJNA308068, PRJEB24031, and PRJEB24829.

## Author Contributions

HZ, ZS, WX, and ZG designed the project. JW performed the research. JW, XM, YH, JY, JS, TT, and ZL analyzed the data and performed the bioinformatics analysis. JW, HZ, ZS, and ZG wrote the manuscript.

## Conflict of Interest

The authors declare that the research was conducted in the absence of any commercial or financial relationships that could be construed as a potential conflict of interest.

## Footnotes

1 https://www.arabidopsis.org/

2 https://phytozome.jgi.doe.gov/pz/portal.html

3 http://molevol.cmima.csic.es/castresana/Gblocks_server.html

4 http://rattan.bamboogdb.org/

## Abbreviations

4CL: 4-coumarate:CoA ligase
C3H: *p*-coumarate 3-hydroxylase
C4H: cinnamate 4-hydroxylase
CAD: cinnamyl alcohol dehydrogenase
CCoAOMT: caffeoyl-CoA *O*-methyltransferase
CCR: cinnamoyl-CoA reductase
COMT: caffeic acid *O*-methyltransferase
CPM: clique percolation method
CSE: caffeoyl shikimate esterase
F5H: ferulate 5-hydroxylase
FDR: false discovery rate
FPKM: fragments per kilobase of transcript per million mapped reads
GABR: Genome Atlas of Bamboo and Rattan project
GO: gene ontology
GSEA: gene set enrichment analysis
HCT: *p*-hydroxycinnamoyl -CoA:quinate/shikimate *p*-hydroxycinnamoyltransferase
ICBR: International Center for Bamboo and Rattan
KEGG: Kyoto Encyclopedia of Genes and Genomes
LHCA: photosystem I light-harvesting complexes
LHCB: photosystem II light harvesting complex gene
MR: mutual rank
PAL: phenylalanine ammonia-lyase
PCC: Pearson’s correlation coefficient
RBH: reciprocal best hit
SCW: secondary cell wall
SD: standard deviation.
