# Supplementary figures and images for: Coexpression Analysis Reveals Dynamic Modules Regulating the Growth and Development of Cirri in the Rattans (Calamus simplicifolius and Daemonorops jenkinsiana)
Source: Front Genet. 2020 May 12;11:378. doi: 10.3389/fgene.2020.00378 (PMC7236543; doi:10.3389/fgene.2020.00378)

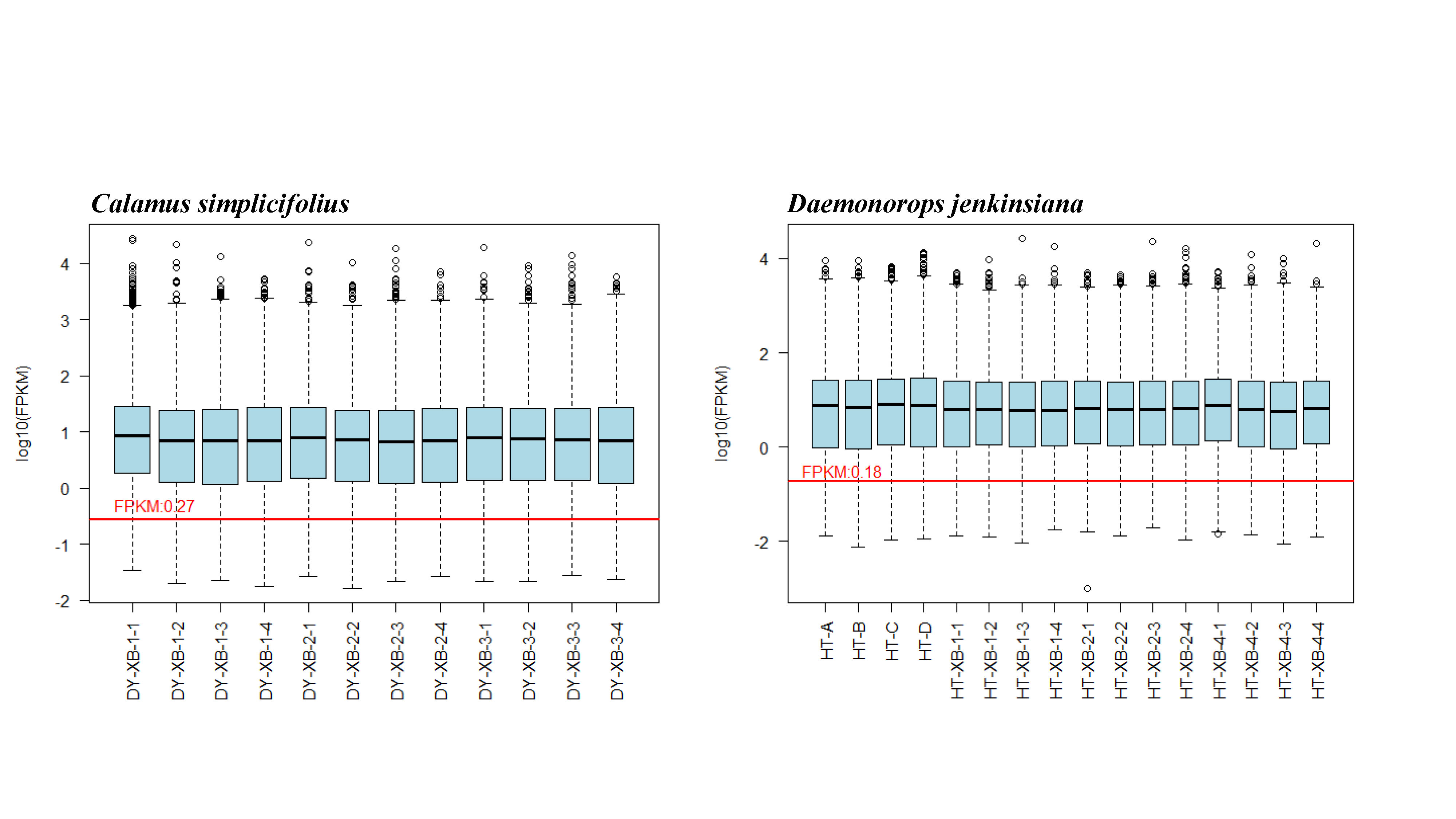

Supplement: Supplementary file 1 [file Image_1.TIF]

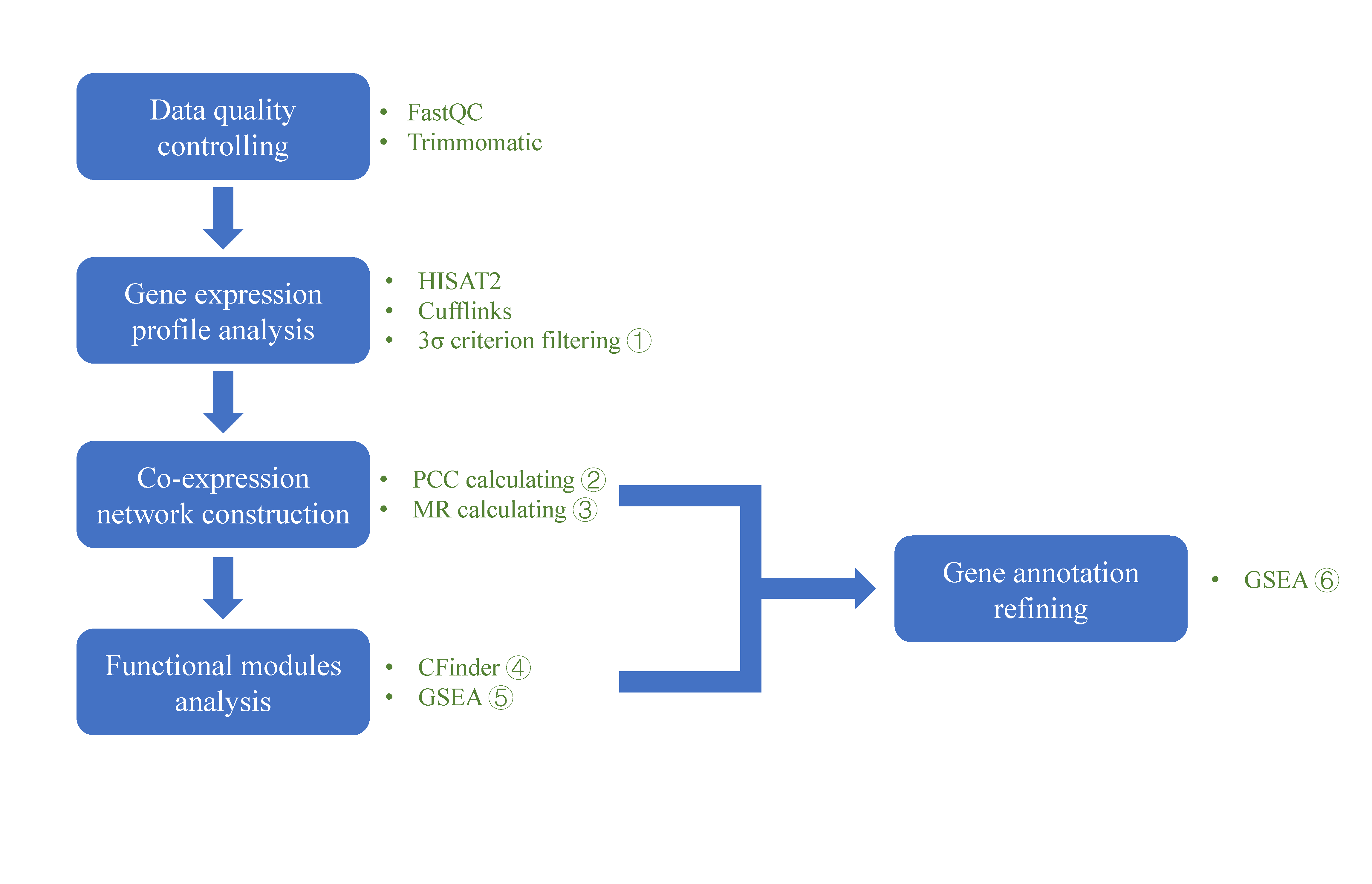

Supplement: Supplementary file 2 [file Image_2.TIF]

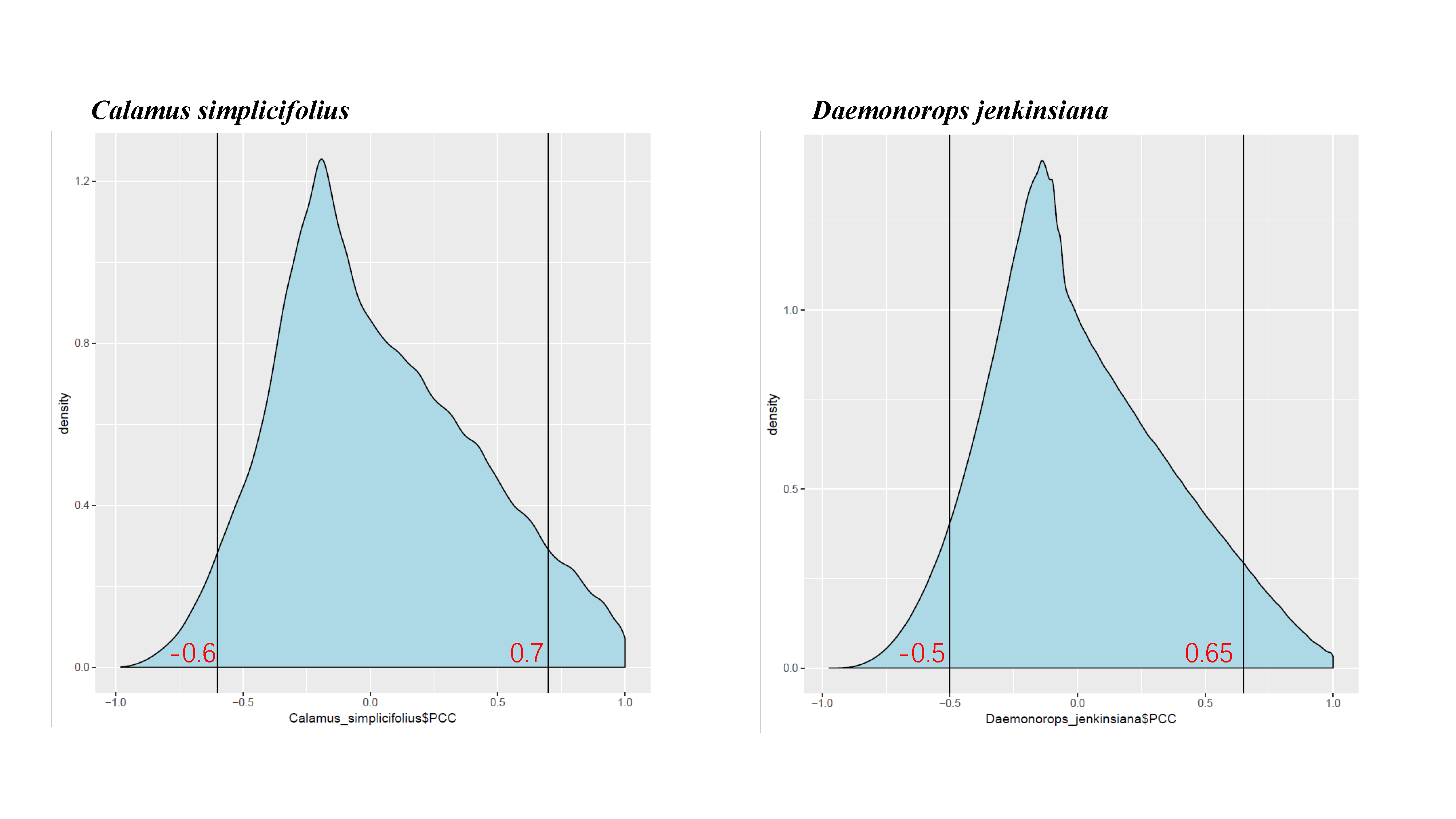

Supplement: Supplementary file 3 [file Image_3.TIF]

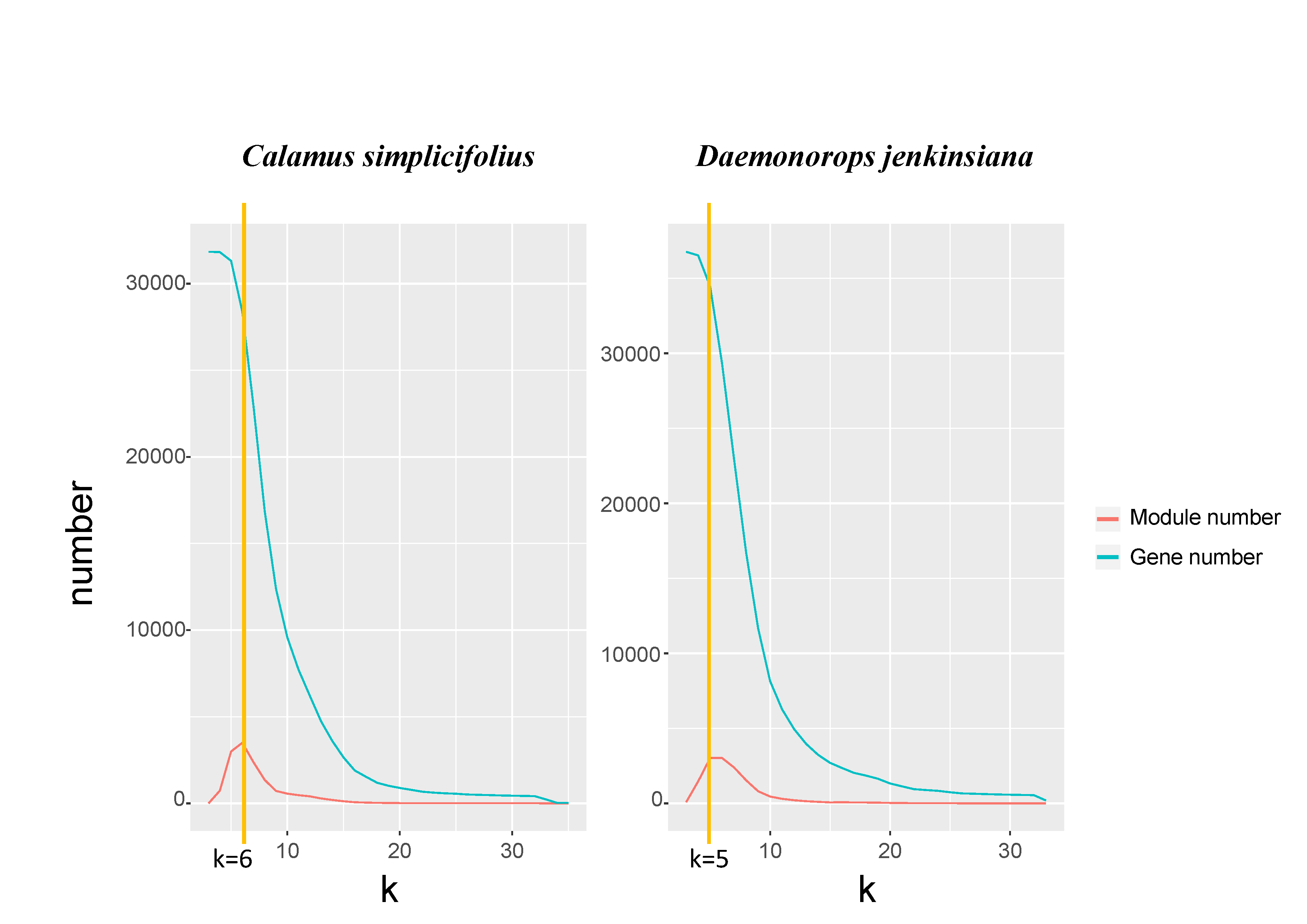

Supplement: Supplementary file 4 [file Image_4.TIF]

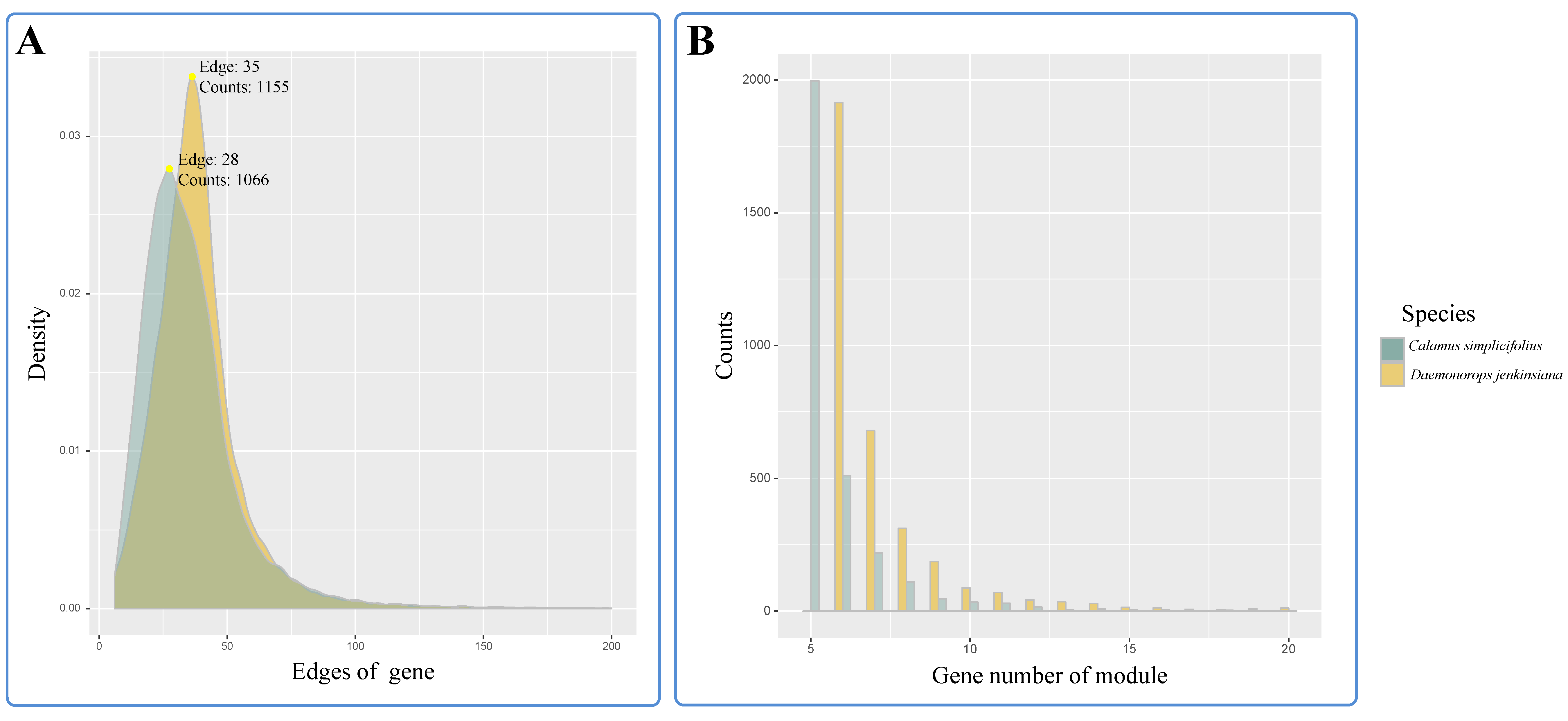

Supplement: Supplementary file 5 [file Image_5.TIF]

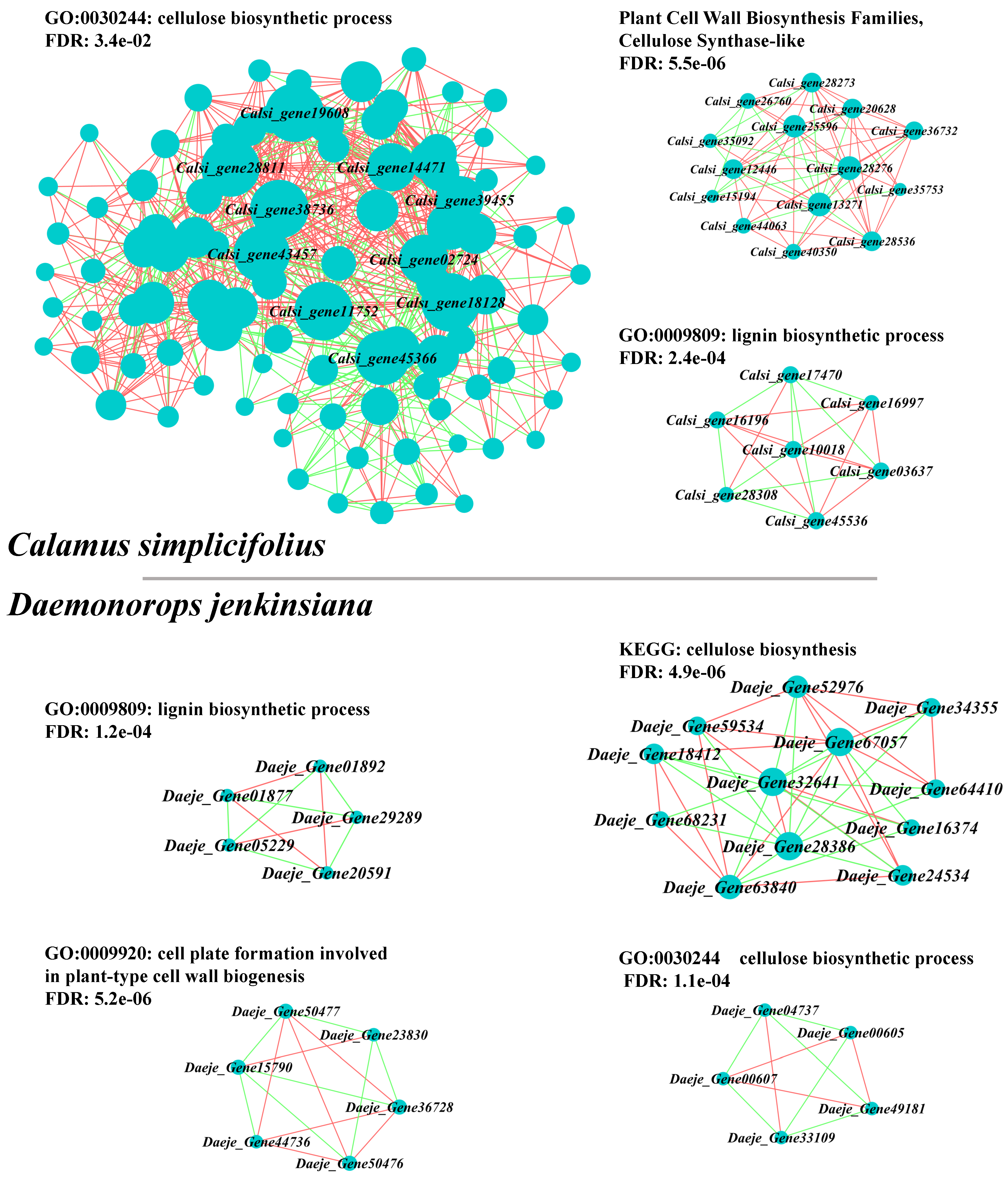

Supplement: Supplementary file 6 [file Image_6.TIF]

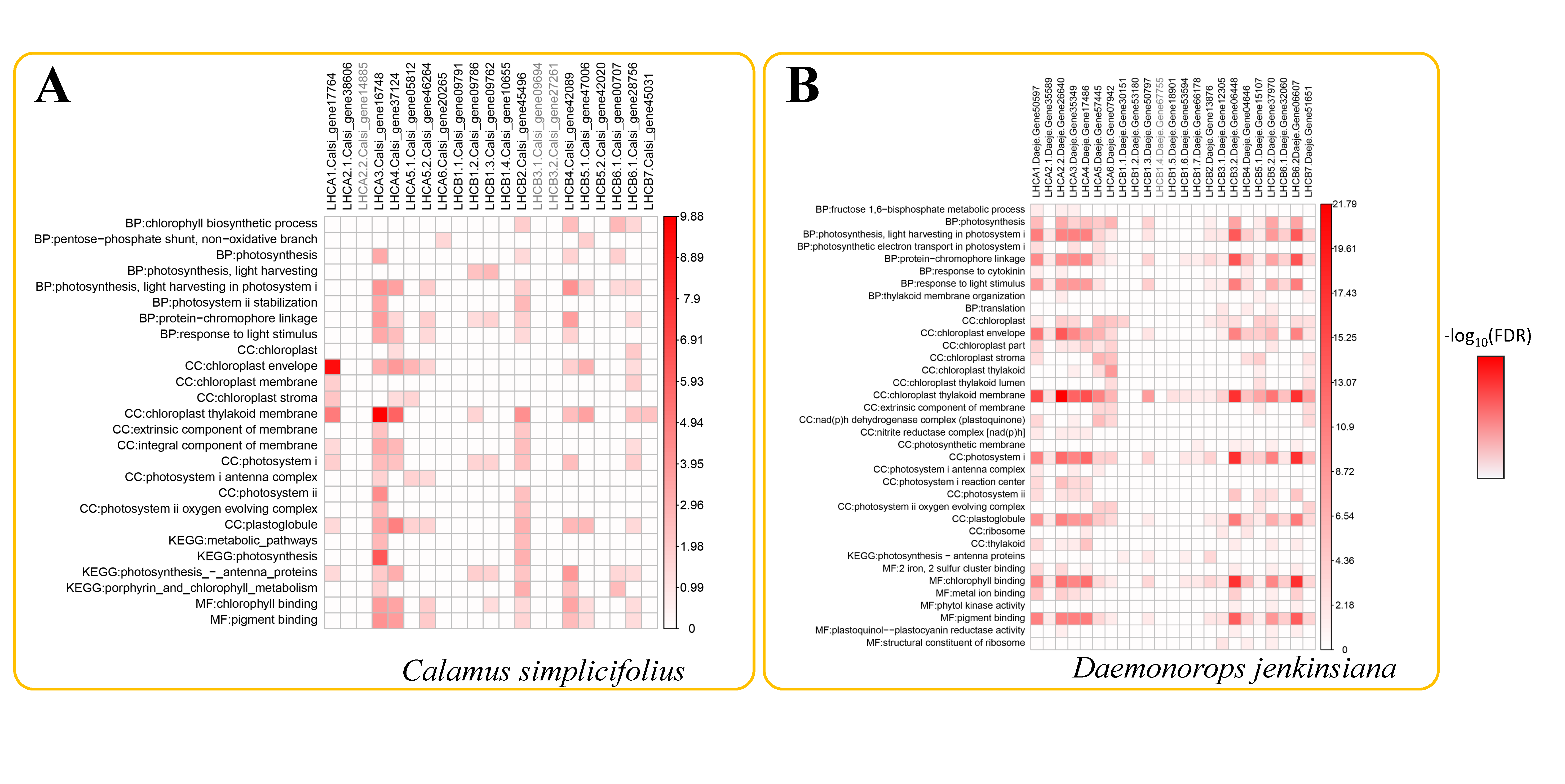

Supplement: Supplementary file 7 [file Image_7.TIF]
