# Supplementary material for: Coexpression Analysis Reveals Dynamic Modules Regulating the Growth and Development of Cirri in the Rattans (Calamus simplicifolius and Daemonorops jenkinsiana)
Source: Front Genet. 2020 May 12;11:378. doi: 10.3389/fgene.2020.00378 (PMC7236543; doi:10.3389/fgene.2020.00378)
Supplement: Supplementary file 9 [file Data_Sheet_1.docx]

**Supplementary figure S1. Box plots of log_10_(FPKM) of samples in the rattans.**

In this study, 0.27 and 0.18 are minimum FPKMs of *Calamus simplicifolius* and *Daemonorops jenkinsiana* respectively.

**Supplementary figure S2. A pipeline figure of co-expression construction and application in the study.**

Labels: ①, The FPKM filtering in here complied the 3σ criterion formula of “threshold = average (5% value) +3 * SD”. The genes whose FPKM less than the threshold were removed for the insignificance of correlation coefficients in low expression genes. FPKM = 0.27 and FPKM = 0.18 were identified the threshold values in Calamus simplicifolius and Daemonorops jenkinsiana, respectively. ②, PCC values of gene pairs were calculated. Meanwhile, we removed weakly correlated gene pairs and only those with strong correlation were reserved. ③, MR values of genes based on PCC were calculated. In the same time, ROC curve analysis was applied to MR threshold selection. According to ROC curve, co-expression gene pairs with a PCC unidirectional rank less than 3 (Rank[A →B] or Rank[B→A]) and an MR value less than 30, were collected for the co-expression network. In this step, the co-expression networks were constructed. ④, We use CFinder to identify modules. Total module nodes and module numbers were taken into account for gene coverage and module number reasonably maximizing. In our study, k = 6 and k = 5 were selected as parameters for CFinder in Calamus simplicifolius and Daemonorops jenkinsiana, respectively. ⑤, GSEA was applied to annotate modules and exclude the suspected modules with FDR > 0.05. ⑥, GSEA was conducted in co-expression genes of each gene to refine gene functional annotation. Firstly, the genes belonging to the modules were annotated with the module functional annotations (FDR < 0.05). Secondly, we gene, and the results with an FDR < 0.05 were recognized as refined annotations.

**Supplementary figure S3. The density map of PCC values of gene pairs in the rattans.**

The left and right figures showed the distribution of PCC values of gene pairs in *C. simplicifolius* and *D. jenkinsiana* respectively. The highest 5% PCC threshold is -0.6 and the lowest 5% PCC threshold is 0.7 in *C. simplicifolius*. The highest 5% PCC threshold is -0.5 and the lowest 5% PCC threshold is 0.65 in *D. jenkinsiana*.

**Supplementary figure S4. The module number and gene number curve of different k value in two rattans.**

The curves were the gist of k value, the parameter of CFinder, selecting in ours job. For gene coverage and module number maximizing reasonably, the k = 6 and k = 5 were selected in *C. simplicifolius* and *D. jenkinsiana* respectively.

**Supplementary figure S5. Statistics of the co-expression networks and functional modules in the rattans.**

(a) Density map of edge number of gene in the rattans. The gene whose edge number > 200 had been shield for low counts and intuition.

(b) Distribution of gene number in the modules. The module whose gene number >20 had been shield for low counts and intuition.

**Supplementary figure S6. Functional regulatory modules related to cell wall biosynthesis modules in the rattans.**

The functional annotations of modules were predicted by GSEA and filtered with FDR < 0.05. The green lines and red lines show negative and positive co-expression relationships between gene pair respectively. The size of node represents edge number linking to the node.

**Supplementary figure S7. The GSEA results of co-expression genes of *LHC* genes in *C. simplicifolius* and *D. jenkinsiana*.**

Gene names and functional terms are displayed in abscissa and ordinate separately. Color dot showed the functional term was enriched in co-expression genes of the *LHC* gene. The functional terms overlapped by distinct *LHC* genes were showed. Grey gene name showed failed GSEA in co-expression genes.
